# Fibroblast growth factor 8 regulates postnatal development of paraventricular nucleus neuroendocrine cells

**DOI:** 10.1186/s12993-015-0081-9

**Published:** 2015-11-04

**Authors:** Karla M. Rodriguez, Erica L. Stevenson, Courtney E. Stewart, Megan L. Linscott, Wilson C. J. Chung

**Affiliations:** Department of Biological Sciences, School of Biomedical Sciences, Kent State University, 53 Cunningham Hall, Kent, OH 44242 USA; School of Biomedical Sciences, Kent State University, Kent, OH 44242 USA

**Keywords:** Fibroblast growth factor 8, Corticotropin-releasing hormone, Vasopressin, HPA-axis, Stress

## Abstract

**Background:**

Fibroblast growth factors (FGFs) are crucial signaling molecules that direct the development of the vertebrate brain. FGF8 gene signaling in particular, may be important for the development of the hypothalamus–pituitary–adrenal (HPA)-axis. Indeed, newborn *Fgf8* hypomorphic mice harbor a major reduction in the number of vasopressin (VP) neurons in the paraventricular nucleus (PVN), the central output component of the HPA-axis. Additionally, recent studies indicated that adult heterozygous (^+/neo^) *Fgf8* hypomorphic mice exhibit more anxiety-like behaviors than wildtype (WT) mice. These studies led us to investigate whether *Fgf8* hypomorphy abrogated VP and/or corticotropin-releasing hormone (CRH) neuronal development in the postnatal day (PN) 21 and adult mouse PVN. Furthermore, we studied whether *Fgf8* hypomorphy disrupted HPA responsiveness in these mice.

**Methods:**

Using immunohistochemistry, we examined the development of VP and CRH neurons located in the PVN of PN 21 and adult *Fgf8*^+/neo^ mice. Moreover, we used a restraint stress (RS) paradigm and measured corticosterone levels with enzyme immunoassays in order to assess HPA axis activation.

**Results:**

The number of VP neurons in the PVN did not differ between WT and *Fgf8*^+/neo^ mice on PN 21 and in adulthood. In contrast, CRH immunoreactivity was much higher in *Fgf8*^+/neo^ mice than in WT mice on PN 21, this difference was no longer shown in adult mice. RS caused a higher increase in corticosterone levels in adult *Fgf8*^+/neo^ mice than in WT mice after 15 min, but no difference was seen after 45 min.

**Conclusions:**

First, *Fgf8* hypomorphy did not eliminate VP and CRH neurons in the mouse PVN, but rather disrupted the postnatal timing of neuropeptide expression onset in PVN neurons. Second, *Fgf8* hypomorphy may, in part, be an explanation for affective disorders involving hyperactivity of the HPA axis, such as anxiety.

## Background

Traditionally, fibroblast growth factor (FGF) signaling molecules are seen as crucial factors that direct the development of the vertebrate brain [1]. However, recent studies using genome-wide approaches on postmortem human brain tissue showed that patients suffering from major depressive disorder (MDD) contain significant alteration in FGF gene expression, when compared to control patients. For instance, FGF1, FGF2, FGF receptor (FGFR) 2 and FGFR3 were significantly down-regulated in the frontal cortex of MDD patients, which strongly suggests that FGF signaling must be disrupted in mood disorders [2, 3]. These data suggest that FGF function may not be limited to just embryonic or perinatal brain development, but extend well into the adult brain. Data from numerous clinical and basic studies have indicated that the hypothalamus–pituitary–adrenal (HPA) axis function exhibits major disruptions in affective disorders during adulthood. For instance, a recent study in middle-aged and aged MDD patients showed higher cortisol levels in the morning compared to aged control patients, which suggests hyperactivity of the HPA axis [4]. Together, these observations led us to infer that the FGF signaling system may play a role in the modulation of HPA function.

The activation of the HPA axis is controlled centrally by corticotropin-releasing hormone (CRH), located in neurons found within the parvocellular subdivision of the paraventricular nucleus (PVN) of the hypothalamus. These CRH neurons project their axons to the median eminence [5–9], which upon a stressful stimulus will release the CRH peptide into the hypophyseal portal vein system to stimulate pituitary adrenocorticotropic hormone release into the general circulation, which then causes the release of the adrenal glucocorticoids in order to maintain body homeostasis [10]. Additional studies show that vasopressin (VP) has a supportive role in regulating the HPA axis [11]. Indeed, VP, co-expressed in CRH neurons in the PVN, potentiates CRH activity at the level of the pituitary corticotroph within the HPA axis, which in turn can lead to anxiety-like behaviors [9, 12, 13].

Recent studies using *Fgf8* hypomorphic mice showed that the adult heterozygous (^+/neo^) *Fgf8* hypomorphic mice exhibited higher levels of anxiety than their wild-type (WT) counterparts [14, 15]. These behavioral studies were correlated with a reduction in the number of tryptophan hydroxylase (Tph; the rate-limiting enzyme for serotonin biosynthesis) expression in the dorsal raphe nucleus *Fgf8*^+/neo^ mice when compared to WT mice [15]. Moreover, a follow-up study showed that the *Fgf8*^+/neo^ dorsal raphe nucleus serotonergic neurons exhibited more c-FOS activation following restraint stress as compared to WT mice [14, 15]. Together, these studies showed that the adult serotonergic function was compromised in *Fgf8*^+/neo^ mice, thus leading to the possibility that FGF8 has the potential to affect the normal development of VP and CRH neurons individually.

Previous studies showed that FGF8 is a morphogen that regulates the normal development of the hypothalamic neuroendocrine cells [16–20]. For example, *Fgf8*^+/neo^ and *Fgf8*^neo/neo^ newborn mice harbor approximately 20–40 % less VP neurons in the PVN as compared to WT littermates [16]. Similarly, FGF8 signaling was important for the embryonic and perinatal development of other hypothalamic neuroendocrine cells, such as the oxytocinergic (OXT), gonadotropin-releasing hormone, and kisspeptinergic neurons [16, 18–20]. However, these studies did not investigate whether the deficiency in FGF8 expression also abrogated VP and/or CRH neuronal development in the young and adult mouse PVN, which represents the central output component of the HPA-axis. To answer this question, we first examined the effects of a deficiency in FGF8 signaling on the VP and CRH neuronal population in postnatal day PN 21 and adult PVN of *Fgf8* hypomorphic mice. Second, we assessed whether HPA-axis function in adult *Fgf8* hypomorphic mice was compromised by examining corticosterone response following restraint stress.

## Methods

### Transgenic animals

Adult 129P2/OlaHsd*CD-1 (obtained from Mouse Regional Resource Centers) [17] male *Fgf8*^+/neo^ x female *Fgf8*^+/neo^ mice were timed-bred in our animal facility on a 12L: 12D cycle with access to food and water ad libitum. All animal procedures were approved by the Institutional Animal Care and Use Committee at Kent State University. Postnatal day (PN) 21 and adult (6–8 weeks) studies were conducted in male WT or *Fgf8*^+/neo^ mice. *Fgf8*^neo/neo^ mice die shortly after birth [17, 18]. *Fgf8* hypomorphic mice contain a neomycin-resistance element inserted into the non-coding region of the *Fgf8* gene, which contains cryptic splice sites that cause an approximately 55 % reduction in functional *Fgf8* mRNA levels in *Fgf8*^neo/neo^ mice [17]. Animals were genotyped using PCR for *Fgf8* (F5′-AAGGGAACAGAGATTTGATG-3′ and R5′-AGTCCACACCACCTCTCAAG-3′), and neomycin (N2/F2) (F5′-GATATTGCTGAAGAGCTTGGC-3′ and R5′-GGTCTCCACAATGAGCTTC-3′) [17].

### Brain tissue collection

Brain tissue from PN 21 and adult mice were collected after euthanasia through decapitation, and were immersion-fixed in 4 % paraformaldehyde/0.1 M phosphate buffer overnight and stored in 30 % sucrose prior to coronal sectioning with a cryostat (Leica CM 1950, Buffalo Grove, IL). PN 21 and adult brain tissue was sectioned at 45 μm in series of four.

### Immunohistochemistry

Brain tissues from PN 21 (one of four series) and adult (one of four series) WT and *Fgf8*^+/neo^ mice were simultaneously processed. The staining conditions were standardized for each of the primary antibodies. These measures were taken in order to minimize the variability. Sections were incubated in 1 % hydrogen peroxide in TBS solution for 15 min (min) at room temperature, washed in TBS, 3 × 5 min on a 2D rotator, and incubated in primary rabbit polyclonal anti-CRH (1:15,000; PA1-37499, Thermo Fisher Scientific, Rockford, IL), rabbit polyclonal anti-VP (1:6000 [19, 20] ) or rabbit polyclonal anti-c-FOS (1:8000; Santa Cruz Biotechnologies, Santa Cruz, CA) diluted in TBS/0.3 % Triton-X (Fisher Scientific, Pittsburgh, PA) and 2 % normal goat serum for 2 days at 4 °C. Sections were washed and incubated with a biotinylated-goat anti-rabbit (1:600) for 2 h at room temperature followed by ABC (1:800) (Vector Laboratories, Burlingame, CA) in TBS for 2 h at room temperature, and reacted with 0.05 % diaminobenzidine + 0.1 % nickel ammonium sulfate (Sigma-Aldrich, St. Louis, MO)/0.01 % H_2_O_2_ in TBS for 20 min. Following the color reaction, sections were dehydrated with increasing percentages of ethanol, and cleared with xylene prior to coverslipping with DPX (Merck, Billerica, MA.)

### Image analysis

#### VP and c-FOS cell number

*VP.* Gray scale digital images of PN 21 (WT: n = 7, HET: n = 4) and adult (WT: n = 4, HET: n = 4) sections through the rostral-caudal extend of the PVN were captured using a 10 × objective mounted on a Olympus microscope fitted with a color camera (SC30, Olympus, Corporation of the Americas, Center Valley, PA) connected to a PC computer. The distance between each immunostained section was 180 μm, which is significantly larger than the average PVN cell size of less than 70 μm [21]. Total number of VP-IR neurons through the rostral-caudal bilateral extend of the PVN was quantified manually by an investigator without knowledge of the genotype of the animals. Only VP-IR neurons with a clearly visible nuclear compartment were included in our analysis. These criteria increased the stringency of our counts, and helped minimize double counts.

*c*-*FOS.* Gray scale digital images of adult (WT: n = 4, HET: n = 4) sections through the rostral-caudal extend of the PVN were captured using a 10 × objective mounted on a Olympus microscope fitted with a color camera (SC30, Olympus, Corporation of the Americas, Center Valley, PA) connected to a PC computer. The distance between each immunostained section was 180 μm. Total number of c-FOS neurons through the rostral-caudal bilateral extend of the PVN was quantified manually by an investigator without knowledge of the genotype of the animals. Only c-FOS-IR neurons with a clearly visible nuclear compartment and nucleoli were included in our analysis. These criteria increased the stringency of our counts, and helped minimize double counts.

#### CRH density

Three rostral-caudal PN 21 (WT: n = 4, HET: n = 4) and adult (WT: n = 4, HET: n = 4) sections per individual animal, representative of the PVN, were matched using the fornix and optic chiasm as anatomical landmarks [22]. The distance between each section was 180 μm. These three grayscale digital images of the PVN per animal were captured using a 4 × objective mounted on a Olympus microscope fitted with a color camera (SC30, Olympus, Corporation of the Americas, Center Valley, PA) connected to a PC computer. The images were analyzed with Cell Sens (Olympus Corporation of the Americas, Center Valley, PA). We generated a standardized threshold mask which accurately covered the CRH immunoreactivity in WT mice. This threshold mask (PN 21 = 179 grayscale value; adult = 127 grayscale value) was then used to quantify bilaterally the CRH-IR density as immunoreactivity covered by pixels in a fixed rectangle (PN 21 = 567,490 μm^2^; adult = 470,384 μm^2^). This method was choosing because we were not able to reliably discern the individual CRH-IR neurons. Furthermore, this method has been applied in past publications [23, 24]. Other methods of protein quantification, such as Western blotting, where also considered however not used for the following major reason: isolation and homogenization of the PVN would have meant the loss of precise anatomical localization of AVP and CRH neurons.

#### Restraint stress

In order to test HPA-axis responsiveness, adult male mice were randomly assigned to either no stress (NS) or restraint stress (RS) groups. Each group experienced the same testing procedures, except the NS mice were left in their cages, while the RS group were removed and restrained in ventilated restraint cones for 15 or 45 min.

#### Corticosterone enzyme-linked immunoassay

Corticosterone levels in adult mice were measured immediately following 15 min of NS or RS condition (WT: n = 5 NS, n = 5 RS; HET: n = 5 NS, n = 5 RS) and 45 min of NS or RS (WT: n = 4 NS, n = 4 RS; HET: n = 4 NS, n = 4 RS). Their trunk blood collected into heparinized tubes, spun at 3000 rpm to collect plasma, and stored at −20 °C until the measurement of corticosterone levels by a commercial enzyme-linked immunoassay kit (ADI-900-097, Enzo Life Sciences) according to manufacturer’s instructions. The intra- and inter-assay coefficients of variation were 6.6–8.4 and 7.8–13.1 %, respectively, and the limit of detection was 26.99 pg/mL.

### Statistical analysis

Data were analyzed for significant differences with *t* tests, one-way or two-way analysis of variance (ANOVA) with genotype and/or restraint stress as between subject variables. Student–Newman–Keuls tests were used for post hoc analysis. Differences were considered significant if *p* < 0.05. Animals and treatments were randomized and coded by an independent investigator. All measurements were conducted by an observer without knowledge of the genotype of the animals.

## Results

### VP neurons in the PVN

**Fig. 1:**
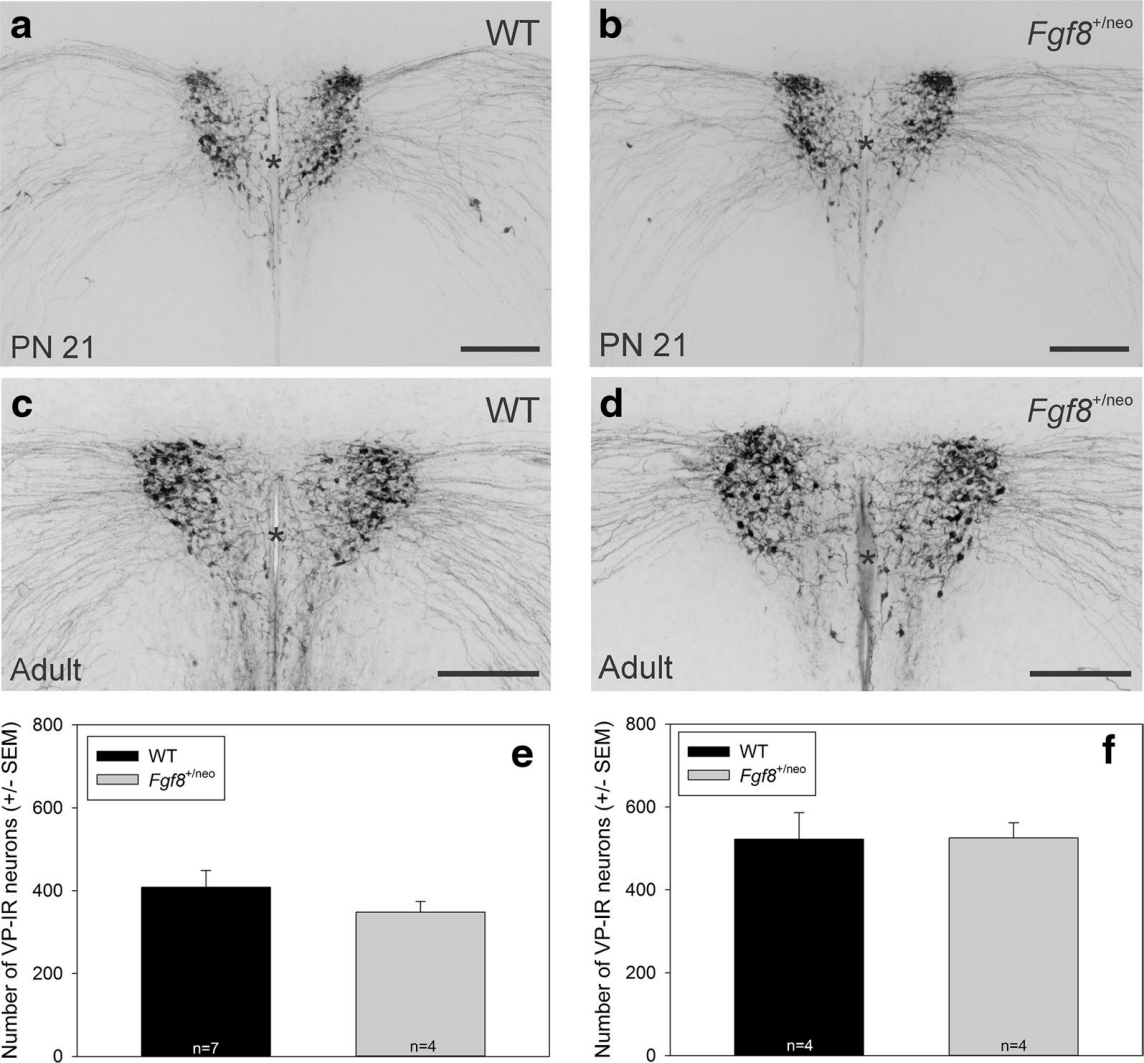
Photomicrographs depicting VP-IR neurons in the PVN of PN 21 **a** WT and **b** *Fgf8* ^+/neo^ mice, as well as adult **c** WT and **d** *Fgf8* ^+/neo^ mice. *Scale bar* is 200 μm. Illustrations visually show no difference between genotypes. *Asterisk* indicates the third ventricle. *Bar graphs* depicting the mean number of VP-IR neurons in the PVN of **e** PN 21 and **f** adult mice. The number of VP-IR neurons did not differ between WT (408.3 ± 40.4 sem) and *Fgf8* ^+/neo^ (348.5 ± 25.3 sem) mice in PN 21 or in adult WT (522.3 ± 63.9 sem) and *Fgf8* ^+/neo^ (525.3 ± 36.7 sem) mice

Two-tailed Student t tests showed that the number of VP neurons did not differ between WT and *Fgf8*^+/neo^ mice in PN 21 (Fig. 1a: t = 1.04, *p* = 0.33) and adults (Fig. 1b: t = −0.04, *p* = 0.97).

### CRH density in the PVN

**Fig. 2:**
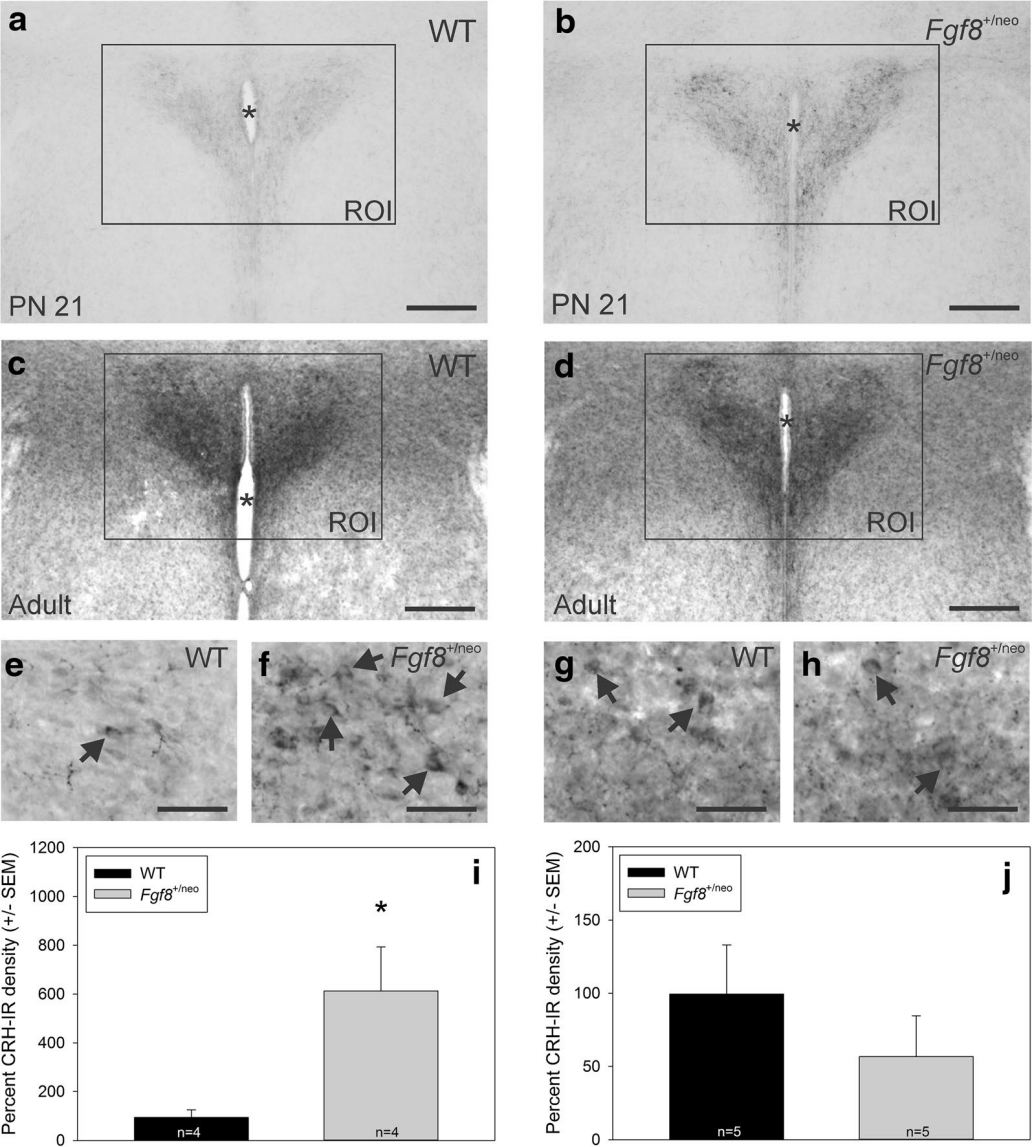
Photomicrographs of CRH immunoreactivity in the PVN of PN21 **a** WT and **b** *Fgf8* ^+/neo^ mice, as well as adult **c** WT and **d** *Fgf8* ^+/neo^ mice. *Scale bar* is 200 μm. The ROI is the region of interest within which we calculated the CRH-IR density. *Asterisk* indicates the third ventricle. Higher magnification photomicrographs of CRH-IR cell bodies (*arrows*) in PN21 **e** WT, **f** *Fgf8* ^+/neo^ and adult **g** WT, **h** *Fgf8* ^+/neo^ mice. *Scale bar* is 50 μm. *Bar graphs* represent PVN CRH-IR density converted into relative percent change compared to the WT group of **i** PN 21 and **j** adult mice. **i** On PN 21, CRH-IR density was significantly lower in WT (94.3 ± 30.6 % sem) compared to *Fgf8* ^+/neo^ (613.0 ± 179.8 % sem) mice. The *asterisk* indicates *p* < 0.05. **j** In adulthood, CRH-IR density did not differ between WT (99.4 ± 33.5 % sem) and *Fgf8* ^+/neo^ (56 ± 27.8 % sem) mice

In PN 21 mice, two-tailed Student *t* tests showed that the CRH density in the PVN was significantly lower in WT than in *Fgf8*^+/neo^ (t = −2.8, *p* < 0.05) (Fig. 2i). In adult mice, two-tailed Student *t* tests showed that CRH density was no longer significant (t = 0.98, *p* < 0.2) (Fig. 2j).

### Corticosteroid levels following restraint stress

**Fig. 3:**
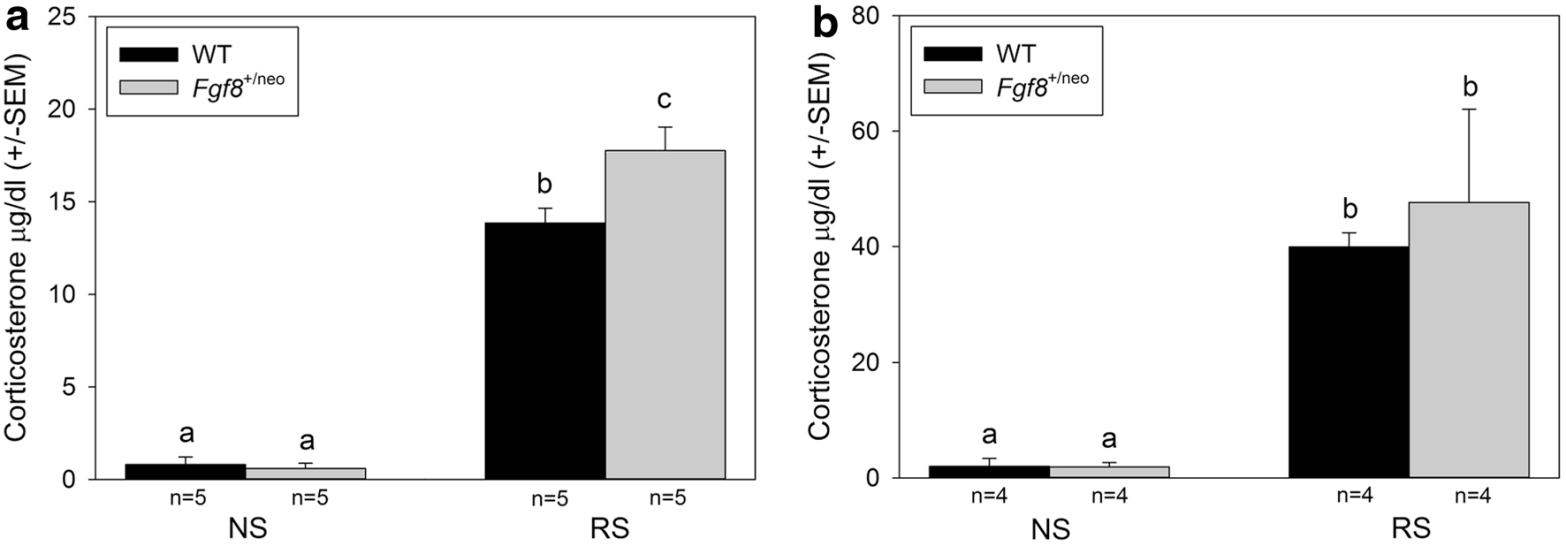
*Bar graphs* depicting corticosterone levels in adult WT and *Fgf8* ^+/neo^ mice in response to RS for **a** 15 or **b** 45 min. **a** RS for 15 min caused a significant rise in corticosteroid levels in WT (13.8 ± 0.8 μg/dL) and *Fgf8* ^+/neo^ mice (17.8 ± 1.3 sem μg/dL) compared to NS WT (0.8 ± 0.4 sem μg/dL) and *Fgf8* ^+/neo^ mice (0.6 ± 0.3 sem μg/dL). The rise in corticosteroid levels after 15 min of RS was significantly lower in WT than in *Fgf8* ^+/neo^ mice. Differences in letters indicate *p* < 0.05.** b** RS for 45 min caused a significant rise in corticosteroid levels in WT (40.0 ± 2.5 μg/dL) and *Fgf8* ^+/neo^ mice (47.7 ± 16.1 sem μg/dL) compared to NS WT (2.0 ± 1.4 sem μg/dL) and *Fgf8* ^+/neo^ mice (1.9 ± 0.7 sem μg/dL). The rise in corticosteroid levels after 45 min of RS did not differ between WT and *Fgf8* ^+/neo^ mice.* Asterisk* indicates *p* < 0.05 between animals treated with NS or RS conditions

*Fifteen minutes*. Two-way analysis of variance showed that RS for 15 min caused a significant increase in circulating corticosterone levels in adult WT and *Fgf8*^+/neo^ mice (Df = 19, F = 364.5, *p* < 0.001). Moreover, there was a significant main genotype effect (Df = 19, F = 5.5, *p* < 0.05), and interaction between restraint and genotype (Df = 19, F = 6.8, *p* < 0.05). Student *t* test analysis showed that the level of corticosterone was much higher in RS than NS WT and *Fgf8*^+/neo^ mice (*p* < 0.001). Furthermore, elevation of circulating corticosterone levels after RS was higher in *Fgf8*^+/neo^ mice than in WT mice (*p* < 0.003) (Fig. 3a).

*Forty*-*five minutes.* Two-way analysis of variance showed that RS stress for 45 min caused a significant increase in circulating corticosterone levels in adult WT and *Fgf8*^+/neo^ mice (Df = 15, F = 24.2, *p* < 0.001). However, there was no significant main genotypic effect (Df = 19, F = 0.6, *p* < 0.4), or interaction between stress and genotype (Df = 15, F = 0.5, *p* < 0.5). Student *t* test analysis showed that the level of corticosterone was much higher in RS than NS WT and *Fgf8*^+/neo^ mice (*p* < 0.001) (Fig. 3b).

### c-FOS neurons in the PVN

**Fig. 4:**
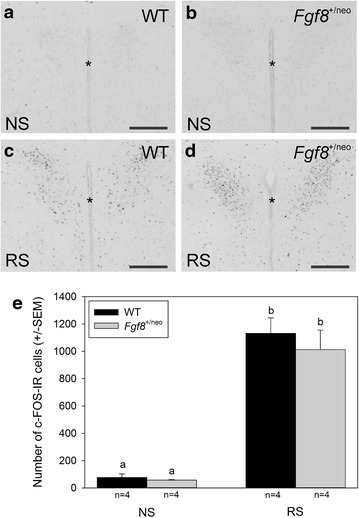
Photomicrographs depicting c-FOS immunoreactivity in adult NS **a** WT and **b** *Fgf8* ^+/neo^ mice and RS **c** WT and **d** *Fgf8* ^+/neo^ mice. *Scale bar* is 200 μm. **e** *Bar graph* depicting the number of c-FOS-IR cells in the adult PVN WT (1131.0 ± 113.3 sem) and *Fgf8* ^+/neo^ (10,212.5 ± 142.6 sem) mice following 45 min of RS, which caused a significant increase in c-FOS-IR PVN cells compared to NS WT (76.0 ± 26.5 sem) and *Fgf8* ^+/neo^ (56.5 ± 3.8 sem) mice (*p* < 0.001). However, two-way ANOVA analysis showed no significant genotypic effect (*p* = 0.5) or interaction between RS and genotype (*p* = 0.6). The difference in letters indicates a significance of *p* < 0.05

Two-way analysis of variance showed that RS for 45 min caused a significant increase in the number of c-Fos-IR in the adult PVN in WT and *Fgf8*^+/neo^ mice (Df = 15, F = 119.4, *p* < 0.001). However, there was no significant main genotypic effect (Df = 19, F = 0.56, *p* = 0.5), or interaction between restraint stress and genotype (Df = 19, F = 0.29, *p* = 0.6) (Fig. 4).

## Discussion

Fibroblast growth factor signaling directs the development of the vertebrate brain [1]. However, *Fgf8* hypomorphy does not only affect neuronal development, but recent studies showed that abrogated FGF expression may have wide-spread neuropathological consequences during adulthood. Here, we showed that *Fgf8* hypomorphy in young and adult mice resulted in changes in postnatal CRH-IR density in the PVN. We found that whilst the CRH-IR density in the PN 21 PVN was much higher in *Fgf8*^+/neo^ mice than in WT mice, this genotype-dependent difference was absent between *Fgf8*^+/neo^ mice and WT mice in adulthood. Moreover, in contrast to newborn mice, the VP-IR neuronal population in the PVN did not differ between WT and *Fgf8*^+/neo^ mice in PN 21 and adulthood [16]. Interestingly, notwithstanding that the VP and CRH immunoreactivity were fully normalized between WT and *Fgf8*^+/neo^ adult mice, we found that adult *Fgf8*^+/neo^ mice mounted a slightly faster, but significantly, corticosterone rise than WT mice in response to RS. From these observations and previous data, we infer that *Fgf8* expression is required for the continued postnatal development/maturation of the VP and CRH neurons in the PVN, and that disruption of this process causes the adult HPA-axis in *Fgf8*^+/neo^ mice to be slightly hyper-responsive when challenged with RS.

Earlier studies found that the number of VP-IR neurons in the PVN was reduced in *Fgf8*^+/neo^ and *Fgf8*^neo/neo^ mice compared to WT mice [16], which may compromise the activation or function of the HPA axis. It has been well-established that *Fgf8* hypomorphy causes abnormal apoptotic cell death during embryonic cortical and olfactory placode development [25, 26], which resulted in permanent morphological and neurochemical changes, such as cortical thinning and the elimination of gonadotropin-releasing hormone neurons [25–27]. Based on these previous data, we hypothesized that the loss of VP-IR neurons found in the newborn *Fgf8* hypomorphic mice [16] was due to the elimination of embryonic PVN neurons that were destined to express VP. This hypothesis led us to predict that the loss of VP-IR neurons in PVN was permanent, and therefore, would be detectable in PN 21 and adult mice. To our surprise, the number of VP-IR neurons did not differ between WT and *Fgf8*^+/neo^ mice in PN 21 or adulthood. These results led us to conclude that *Fgf8* hypomorphy-dependent premature apoptosis in the PVN was not the cause for the reduced number of the VP-IR neurons in the newborn *Fgf8* hypomorphic PVN. The most logical explanation is that the newborn and postnatal *Fgf8*^+/neo^ hypomorphic PVN actually are comprised with a normal complement of neurons that are able to express VP, and that this expression is disturbed by the deficiency in FGF8 signaling. Previous studies showed that oxytocin expressing PVN neurons are similarly reduced in *Fgf8* hypomorphic newborn mice, which has been posited to be due to a disruption of the post-translational processing of the oxytocin prohormone to mature oxytocin [28]. Although, this possibility may have caused the reduction in VP-IR neurons in the newborn PVN, it is far more likely that *Fgf8* hypomorphy interfered VP gene transcription. Indeed, in situ hybridization studies showed a dramatic reduction of VP mRNA expression in the newborn *Fgf8* hypomorphic PVN [16].

In contrast to the VP-IR neurons in the PVN, CRH-IR neuronal density in the PVN was much higher in *Fgf8*^+/neo^ mice as compared to WT mice in PN 21, which was found to be fully normalized between WT and *Fgf8*^+/neo^ mice in adulthood. These data then led us to a reinterpretation of the role FGF8 has in the mammalian brain. FGF8 signaling may remain relevant during postnatal PVN development and/or function. Specifically, we hypothesize that FGF8 signaling may regulate the timing of neuropeptide expression onset in postnatal PVN neurons as supported by the *Fgf8* hypomorphy-dependent delay in VP expression and advancement of CRH expression in postnatal PVN neurons. Currently, more studies are required to fully investigate and explore the validity of this hypothesis.

Although, VP and CRH expression was normalized between WT and *Fgf8*^+/neo^ adult mice, the rise in the corticosterone levels after 15 min of RS in adult *Fgf8*^+/neo^ mice was faster than in WT mice, which was no longer detectable after 45 min of RS. These basic experimental studies are in line with previous behavioral studies suggesting that the HPA axis in *Fgf8*^+/neo^ mice may be hyperactive [14, 15]. Indeed, *Fgf8*^+/neo^ hypomorphic mice exhibited higher levels of anxiety-like behavior, which coincided with a reduction in the number of Tph-expressing neurons in the dorsal raphe nucleus [15]. Interestingly, in a follow-up study *Fgf8*^+/neo^ dorsal raphe nucleus serotonergic neurons exhibited more c-FOS activation following RS as compared to WT mice [14, 15]. These studies showed that the adult serotonergic function was compromised in *Fgf8*^+/neo^ mice, which together with the abnormal postnatal development of the VP and CRH neurons in the PVN may be the underlying cause of *Fgf8* hypomorphy-dependent HPA axis hyperactivity, and increased anxiety-like behaviors.

Fibroblast growth factor-dependent disruption of HPA function may contribute to the onset of other affective disorders, such as MDD. For instance, genome-wide studies in postmortem human brain tissue from patients with MDD showed that they harbored significant abnormalities in FGF expression in the frontal cortex, when compared to control patients [2, 3]. Specifically, FGF1, FGF2, FGF receptor (FGFR) 2 and FGFR3 were down-regulated in the frontal cortex of MDD patients, which strongly suggests that FGF signaling must be disrupted in mood disorders [2, 3]. Based on our studies showing an artificial partial inactivation of FGF8 signaling in the young and adult mouse, we postulate that FGF8 signaling may similarly be involved and individuals with inactivating mutations in *Fgf8* may be predisposed to affective disorders.

## Conclusion

Here, our studies led us to conclude that FGF8 signaling remains very important for the ongoing postnatal development of the PVN in young and adult mice. First, partial inactivation of FGF8 signaling did not lead to the elimination of VP and CRH neurons that reside in the mouse PVN, but rather caused a disruption in the postnatal timing of neuropeptide expression onset in PVN neurons. Second, *Fgf8* hypomorphy may, in part, be an explanation for affective disorders involving hyperactivity of the HPA axis, such as anxiety and MDD.
